# Supplementary material for: Single-cell RNA transcriptome analysis of CNS immune cells reveals CXCL16/CXCR6 as maintenance factors for tissue-resident T cells that drive synapse elimination
Source: Genome Med. 2022 Sep 24;14:108. doi: 10.1186/s13073-022-01111-0 (PMC9509564; doi:10.1186/s13073-022-01111-0)
Supplement: Supplementary file 1 — Additional file 1: Table S1. Genes used to define cluster identity. Table S2. Top 25 defining genes for clusters 2 and 4. Figure S1. Split single cell clustering and nCount_RNA and nFeature_RNA plots. a. Split single cell clustering of the 4 biological samples processed for scRNA-sequencing. b. nCount_RNA and nFeature_RNA plots for cells that remained after QC. c. Representative gating strategy to identify live cells in mock and WNV-infected WT cortical and hippocampal samples (middle and right panel). A fluorescence minus one control (left panel) was also included. Figure S2. Pathway analysis of microglia clusters and tSNEs of microglia and TRM markers. a. GO biological processes pathway analysis using generally applicable gene set enrichment (GAGE) method [36] based on the log2 fold changes from the single cell differential expression analysis. Pathway lists were generated with genes downregulated in Cluster 1 compared to Cluster 0 (top panel) and upregulated in Cluster 1 compared to Cluster 0 (bottom panel). b. Pathway lists were generated with genes downregulated in Cluster 3 compared to Cluster 1. No genes were significantly upregulated in Cluster 3 compared to Cluster 1. c. tSNE plots depicting the relative expression of microglial core genes, P2ry12, Cx3cr1, Fcls, Siglech, Hexb, and Tmem119. d. tSNE plots depicting the relative expression of TRM genes, Itgae, Cd44, Cd69, and Itga1. Color key indicates the expression levels. Figure S3. Characterization of CXCR6+CD4+ and CD8+ T cells during WNV infection. a, c. Flow cytometric analysis of the percent and number of CD4+ T cells that are CXCR6+ in the cortex (a) or hippocampus (c) of WNV-infected WT mice at 7, 25 and 52 DPI. b,d. Flow cytometric analysis of the number of CD8+ T cells that are CXCR6+ in the cortex (b) or hippocampus (d) of WNV-infected WT mice at 7, 25 and 52 DPI. e. Flow cytometric analysis of the percent of CXCR6+ cells that are CD103+NS4B- in the cortex (left) or hippocampus (right) of WNV-infe [file 13073_2022_1111_MOESM1_ESM.docx]

**Additional Files**

Table S1. Genes used to define cluster identity

| Cluster |  | Gene |
| --- | --- | --- |
| 0 | Resting microglia | *P2ry12, Tmem119, Siglech* |
| 1 | Activated microglia -1 | *Cd74, Apoe, Ccl12* |
| 2 | CD8+ T cells | *Lck, Cd8a, Itgae* |
| 3 | Activated Microglia-2 | *Apoe, H2-Aa1, Ctss1* |
| 4 | CD4+ T cells | *Cd247, Cd4, Ctla4* |
| 5 | Macrophages | *Wfdc17, Ms4a4a, Lyz2* |
| 6 | Astrocytes | *Mt3, Fxyd1, Igfbp2* |
| 7 | IEG+ microglia | *Atf3, Fos, Egr1* |
| 8 | B cells-1 | *Igkc, Mzb1, Cd79a* |
| 9 | B cells-2 | *Cd191, Blk, Cd79a1* |

Table S2. Top 25 defining genes for clusters 2 and 4

| Rank | Cluster 2 | Cluster 4 |
| --- | --- | --- |
| 1 | *Cd8b1* | *Cd4* |
| 2 | *Nkg7* | *Cd40lg* |
| 3 | *Cd8a* | *Cd3e* |
| 4 | *Gzmb* | *Tnfrsf4* |
| 5 | *Ly6c2* | *Icos* |
| 6 | *Cd3g* | *Cd3d* |
| 7 | *Gzmk* | *Lat* |
| 8 | *Ctsw* | *Thy1* |
| 9 | *S100a6* | *Gimap3* |
| 10 | *Ms4a4b* | *Cd3g* |
| 11 | *Cd3e* | *Tnfrsf18* |
| 12 | *Cxcr6* | *Ms4a4b* |
| 13 | *Thy1* | *Lck* |
| 14 | *Cd3d* | *Cd28* |
| 15 | *Plac8* | *Cd2* |
| 16 | *Gimap4* | *Trbc2* |
| 17 | *Lck* | *Ctla4* |
| 18 | *Lat* | *Cd247* |
| 19 | *Gimap3* | *Gimap4* |
| 20 | *Ptprcap* | *Trac* |
| 21 | *Ctla2a* | *Podnl1* |
| 22 | *Gimap7* | *Ptprcap* |
| 23 | *Klrc1* | *S100a10* |
| 24 | *Jaml* | *Izumo1r* |
| 25 | *Cd2* | *Ltb* |

**
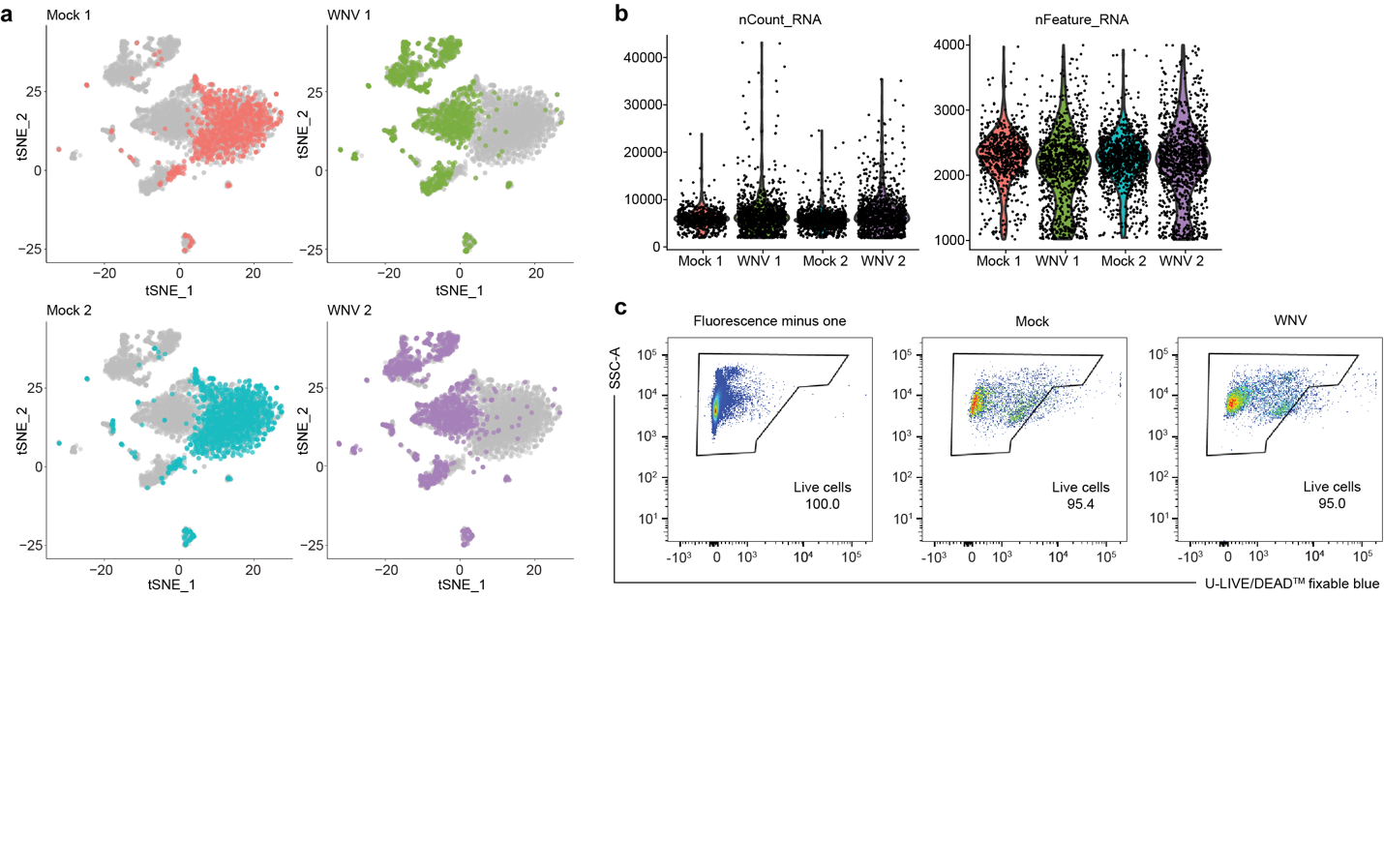
Figure S1. Split single cell clustering and nCount_RNA and nFeature_RNA plots. a.** Split single cell clustering of the 4 biological samples processed for scRNA-sequencing. **b.** nCount_RNA and nFeature_RNA plots for cells that remained after QC. **c.** Representative gating strategy to identify live cells in mock and WNV-infected WT cortical and hippocampal samples (middle and right panel). A fluorescence minus one control (left panel) was also included.

**
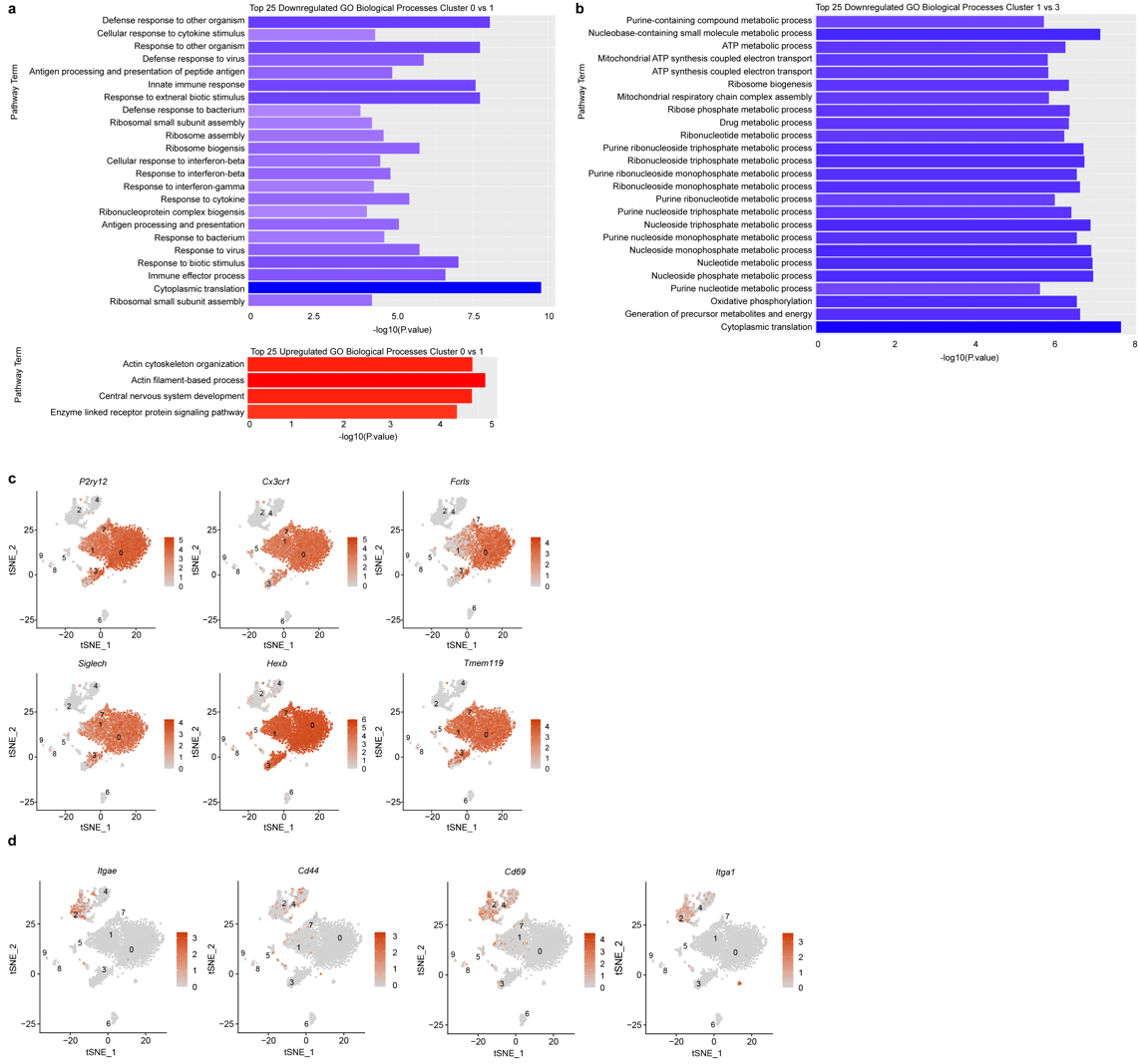
**

**Figure S2. Pathway analysis of microglia clusters and tSNEs of microglia and T_R_M markers. a.** GO biological processes pathway analysis using generally applicable gene set enrichment (GAGE) method^75^ based on the log2 fold changes from the single cell differential expression analysis. Pathway lists were generated with genes downregulated in Cluster 1 compared to Cluster 0 (top panel) and upregulated in Cluster 1 compared to Cluster 0 (bottom panel). **b.** Pathway lists were generated with genes downregulated in Cluster 3 compared to Cluster 1. No genes were significantly upregulated in Cluster 3 compared to Cluster 1. **c.** tSNE plots depicting the relative expression of microglial core genes, *P2ry12, Cx3cr1, Fcls, Siglech, Hexb,* and *Tmem119*. **d.** tSNE plots depicting the relative expression of T_R_M genes, *Itgae, Cd44, Cd69,* and *Itga1*. Color key indicates the expression levels.

**
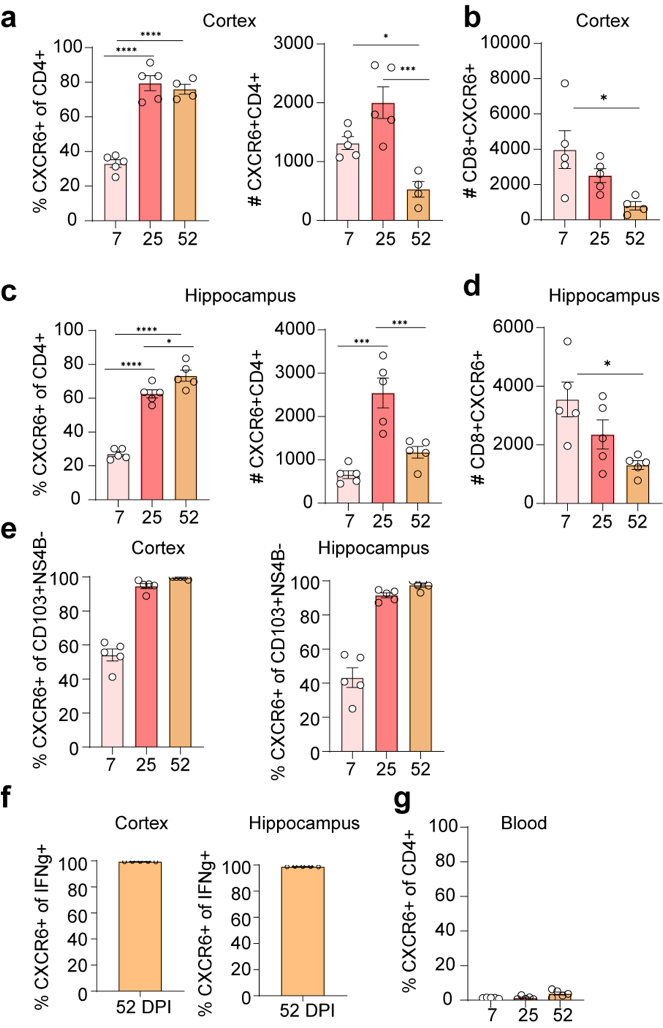
**

**Figure S3. Characterization of CXCR6^+^CD4^+^ and CD8^+^ T cells during WNV infection. a, c.** Flow cytometric analysis of the percent and number of CD4^+^ T cells that are CXCR6^+^ in the cortex (a) or hippocampus (c) of WNV-infected WT mice at 7, 25 and 52 DPI. **b,d.** Flow cytometric analysis of the number of CD8^+^ T cells that are CXCR6^+^ in the cortex (b) or hippocampus (d) of WNV-infected WT mice at 7, 25 and 52 DPI.  **e.** Flow cytometric analysis of the percent of CXCR6^+^ cells that are CD103^+^NS4B^-^ in the cortex (left) or hippocampus (right) of WNV-infected WT mice at 7, 25 and 52 DPI.  **f.** Flow cytometric analysis of the percent of CD8^+^ T cells that are IFNg^+^ in the cortex and hippocampus of WNV-infected WT mice at 52 DPI**. g.** Flow cytometric analysis of the percent of CD4^+^ T cells that are CXCR6^+^ in the blood of WNV- infected mice at 7, 25 and 52 DPI. Data represent the mean±s.e.m. and were analyzed by unpaired Student’s *t*-test.

**
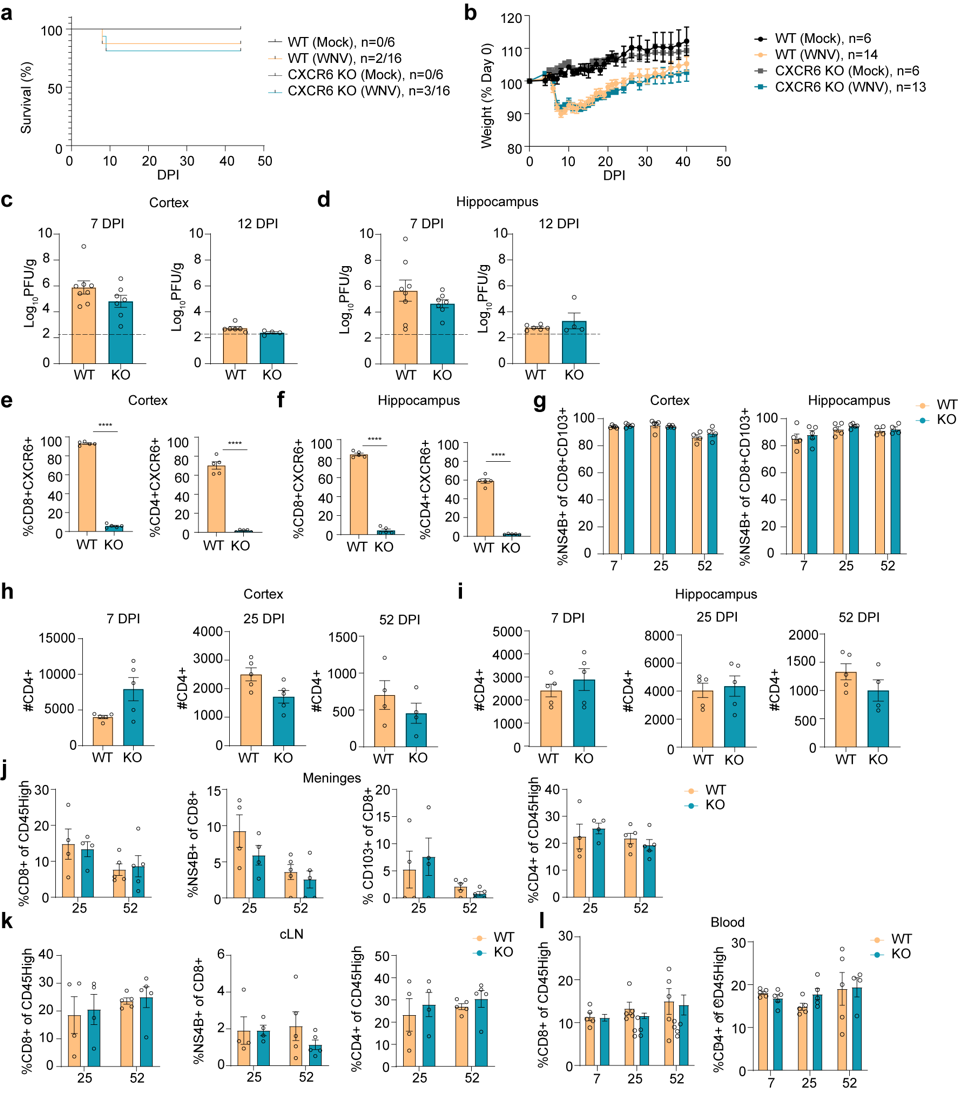
**

**Figure S4. Characterization of *Cxcr6^-/-^* mice. a.** Survival curve of WNV-infected *Cxcr6^-/-^* and WT mice. **b.** Weight loss course in WNV-infected Cx*cr6^-/-^* and WT mice. **c, d.** Viral loads measured by plaque assay in the cortex (c) and hippocampus (d) of WNV-infected *Cxcr6^-/-^* and WT mice at 7 or 12 DPI. **e, f.** Flow cytometric analysis of the percent of CD8^+^CXCR6^+^ and percent of CD4^+^CXCR6^+^ T cells in the cortex (e) and hippocampus (f) of WT and *Cxcr6^-/-^* animals at 25 DPI. **g.** Flow cytometric analysis of the percent of CD8^+^CD103^+^ T cells that are NS4B^+^ in the cortex and hippocampus of WNV-infected WT and *Cxcr6^-/-^* at 7, 25, and 52 DPI. **h,i.** Flow cytometric analysis of the total number of CD4^+^ T cells in the cortex (h) and hippocampus (i) of WT and *Cxcr6^-/-^* animals at 7, 25 and 52 DPI. **j.** Flow cytometric analysis of the percent of CD45^high^ cells that are CD8^+^, percent of CD8^+^ cells that are NS4B^+^ or CD103^+^, and percent of CD45^high^ cells that are CD4^+^ in the meninges of WNV-infected Cx*cr6^-/-^* and WT mice at 25 and 52 DPI. **k.** Flow cytometric analysis of the percent of CD45^high^ cells that are CD8^+^, percent of CD8^+^ cells that are NS4B^+^, and percent of CD45^high^ cells that are CD4^+^ in the cervical lymph nodes (cLN) of WNV-infected Cx*cr6^-/-^* and WT mice at 25 and 52 DPI. **l.** Flow cytometric analysis of the percent of CD45^high^ cells that are CD8^+^ or CD4^+^ in the blood of WNV-infected Cx*cr6^-/-^* and WT mice at 7, 25 and 52 DPI. Data represent the mean±s.e.m. and were analyzed by unpaired Student’s *t*-test. *****P* < 0.0001.

**
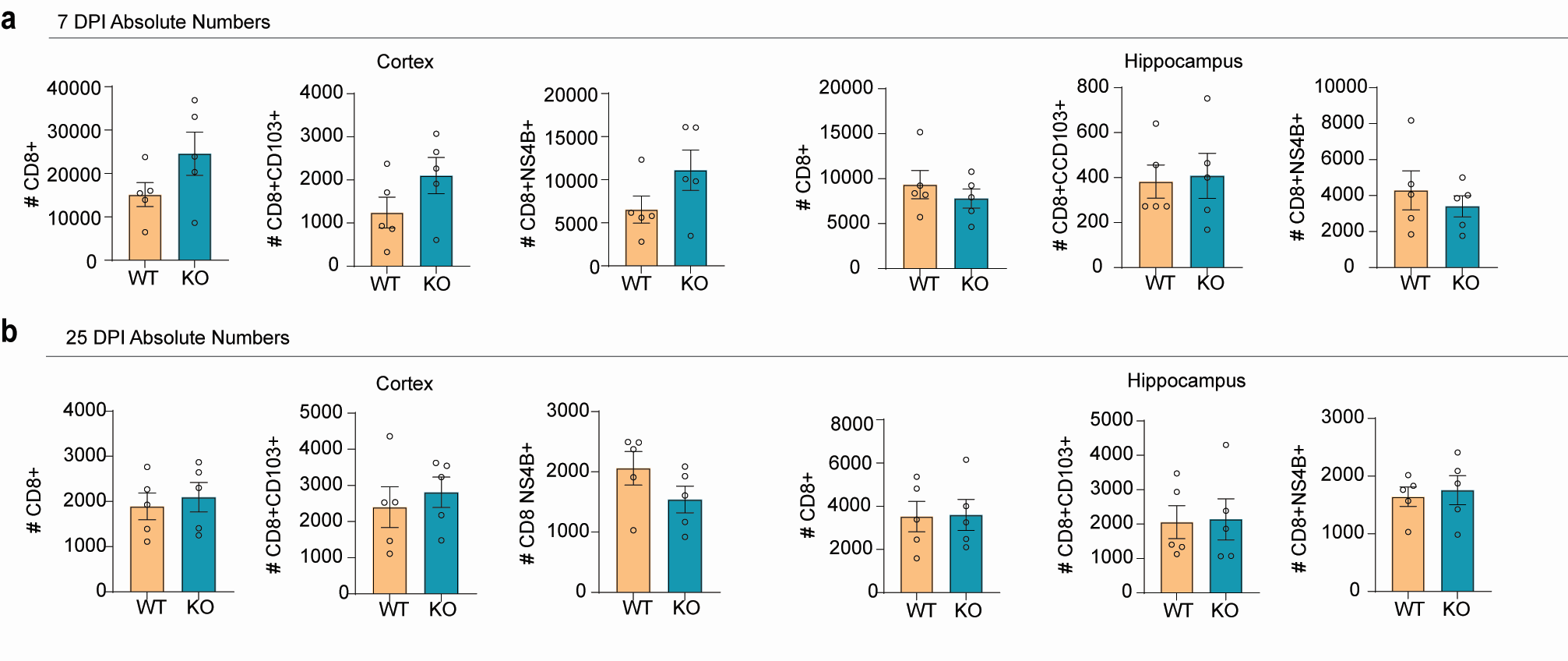
Figure S5. Analysis of T cell numbers in CXCR6-deficient animals. a,b.** Flow cytometric analysis of total numbers of CD8^+^, CD8^+^CD103^+^, and CD8^+^NS4B^+^ cells in the cortex and hippocampus of WT and *Cxcr6^-/-^* mice at 7 (a) or 25 (b) DPI. Data represent the mean±s.e.m. and were analyzed by unpaired

**
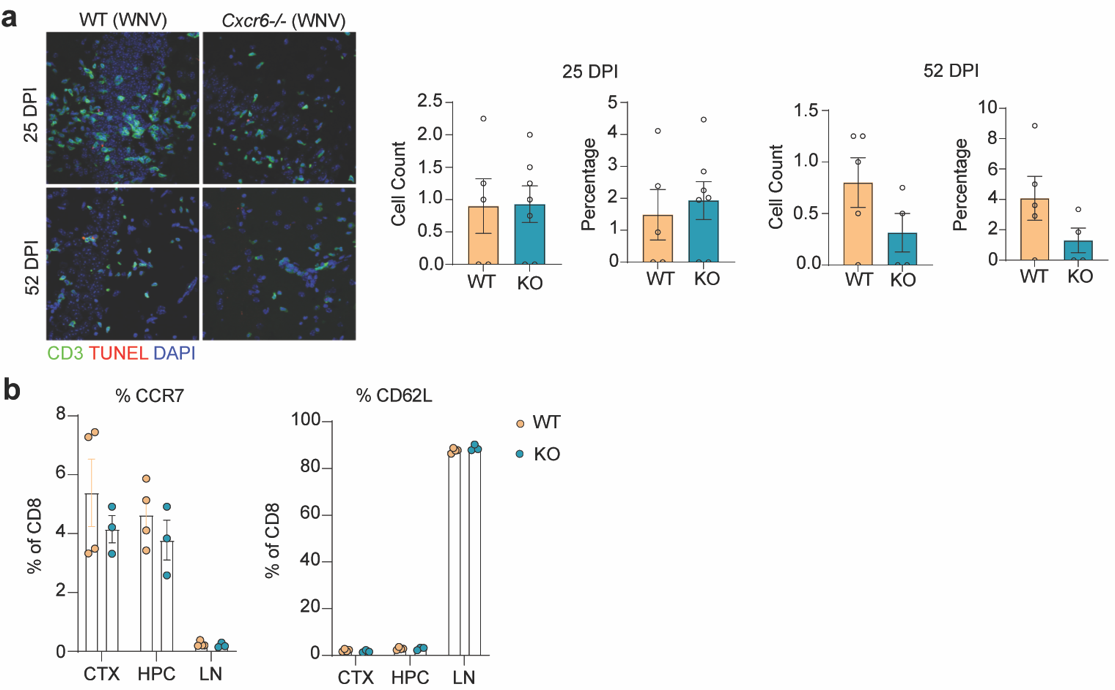
**

**Figure S6.** **Loss of CD8^+^ T cells in the CNS is not due to T cell apoptosis, egression to the lymph node, or accumulation in the meninges in *Cxcr6^-/-^* animals. a.** Representative immunostaining and quantification of TUNEL (red), CD3 (green) and DAPI (blue) in CA3 region of the hippocampus of WNV-infected WT or *Cxcr6^-/-^* mice at 25 and 52 DPI. Cell count quantified by counting of CD3^+^TUNEL^+^ cells per image and percentage quantified by number of CD3^+^TUNEL^+^ cells normalized to total CD3^+^ cells. **b.** Flow cytometric analysis of the percentage of CD8^+^ T cells that are CCR7^+^ or CD62L^+^ in the cortex, hippocampus, and lymph nodes at 35 DPI. Data represent the mean±s.e.m. and were analyzed by unpaired Student’s t-testStudent’s *t*-test.

**
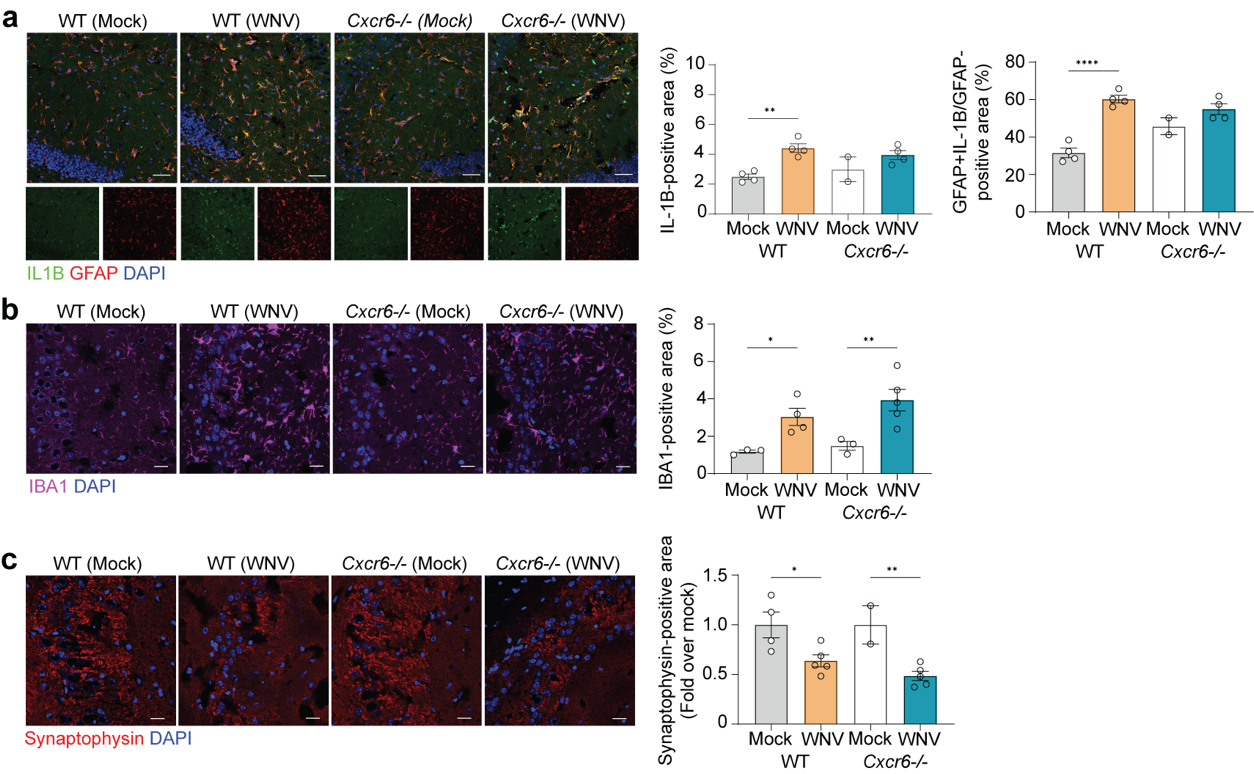
**

**Figure S7. Gliosis persists in the hippocampus of WNV-infected WT and *Cxcr6^-/-^* mice at 25 DPI. a.** Representative immunostaining at 25 DPI of IL-1β (green), GFAP (red) and DAPI (blue) in CA3 region of the hippocampus of mock or WNV-infected WT or *Cxcr6^-/-^* mice, followed by quantification of percent GFAP^+^IL-1β^+^ area, normalized to the total GFAP^+^ area. **b.** Representative immunostaining at 25 DPI of IBA1 in mock- or WNV-infected WT or *Cxcr6^-/-^* mice, showing staining for IBA1 (magenta) and DAPI (blue), followed by quantification of percent IBA1^+^ area in the hippocampus. **c.** Representative immunostaining and quantification of synapses in the CA3 region of the hippocampus in mock- or WNV-infected WT or *Cxcr6^-/-^* animals at 25 DPI showing staining for synaptophysin (red) and DAPI (blue). Synaptophysin quantified by percent positive area. Scale bars, 50 μm (a) or 20 μm (b,c). Data represent the mean±s.e.m. and were analyzed by two-way ANOVA and corrected for multiple comparisons. *P<0.05, **P<0.005, *****P* < 0.0001.

**
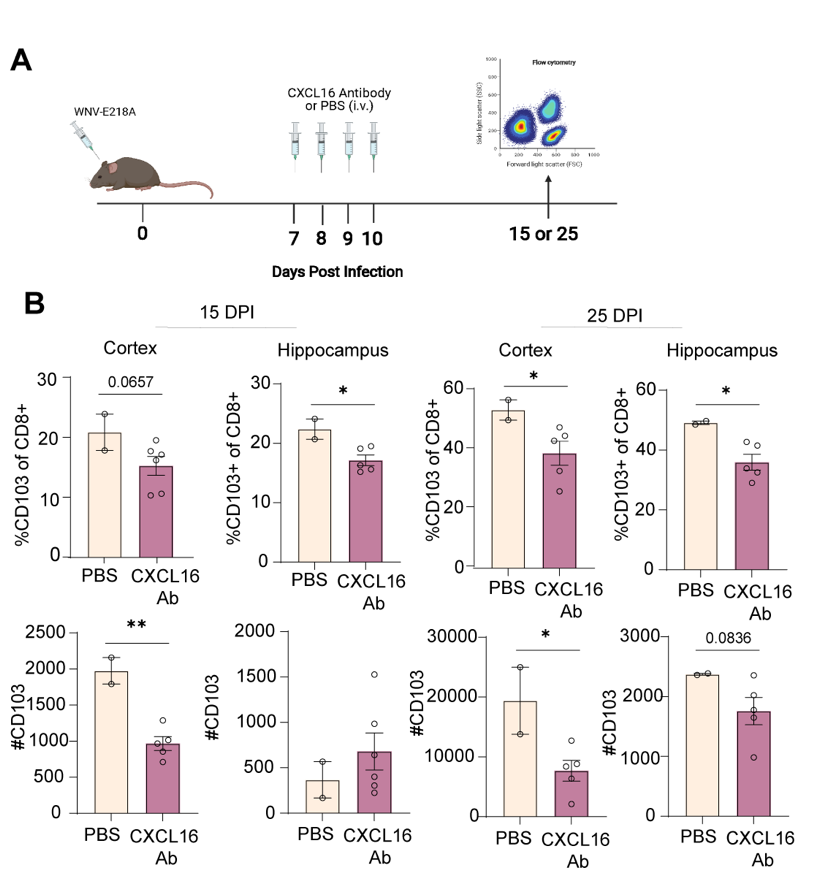
**

**Figure S8. CXCL16 neutralization leads to decreased percentage and numbers of T_R_M cells in the CNS at 15 and 25 DPI. a.** Schematic depicting experimental design for CXCL16 neutralizing antibody experiment. Mice were infected (i.c.) with 1×10^4^ p.f.u. WNV-NS5-E218A and administered CXCL16 antibody or isotype control via retro-orbital injection, and harvested 15 DPI. **b.** Quantification of percentage and number of CD8^+^ cells that are CD103^+^ in the cortex (left) and hippocampus (right) at 15 DPI in mice that received PBS or CXCL16 neutralizing antibody. **c.** Quantification of percentage and number of CD8^+^ cells that are CD103^+^ in the cortex (left) and hippocampus (right) at 25 DPI in mice that received PBS or CXCL16 neutralizing antibody. Data represent the mean±s.e.m. and were analyzed by unpaired Student’s *t*-test. *P<0.05.
